# Imbalance of Essential Metals in Traumatic Brain Injury and Its Possible Link with Disorders of Consciousness

**DOI:** 10.3390/ijms24076867

**Published:** 2023-04-06

**Authors:** Rosanna Squitti, Giuseppe Reale, Vincenzo Tondolo, Daniela Crescenti, Sonia Bellini, Marco Moci, Pietro Caliandro, Luca Padua, Mauro Rongioletti

**Affiliations:** 1 Department of Laboratory Science, Research and Development Division, Fatebenefratelli Isola Tiberina, Gemelli Isola, 00186 Rome, Italy; 2 Fondazione Policlinico Universitario A. Gemelli IRCCS, UOC Neuroriabilitazione ad Alta Intensità Largo Agostino Gemelli 8, 00168 Rome, Italy; 3 Digestive and Colorectal Surgery, Fatebenefratelli Isola Tiberina, Gemelli Isola, 00186 Rome, Italy; 4 Digestive Surgery Unit, Fondazione Policlinico Universitario A. Gemelli IRCCS, Largo Agostino Gemelli 8, 00168 Rome, Italy; 5 Molecular Markers Laboratory, IRCCS Istituto Centro San Giovanni di Dio Fatebenefratelli, 25125 Brescia, Italy; 6 Fondazione Policlinico Universitario A. Gemelli IRCCS, UOC Neurologia, 00168 Rome, Italy

**Keywords:** traumatic brain injury, disorders of consciousness, Alzheimer’s disease, metals, copper, iron, zinc

## Abstract

Dysfunction of the complex cerebral networks underlying wakefulness and awareness is responsible for Disorders of Consciousness (DoC). Traumatic Brain Injury (TBI) is a common cause of DoC, and it is responsible for a multi-dimensional pathological cascade that affects the proper functioning of the brainstem and brain consciousness pathways. Iron (Fe), Zinc (Zn), and Copper (Cu) have a role in the neurophysiology of both the ascending reticular activating system, a multi-neurotransmitter network located in the brainstem that is crucial for consciousness, and several brain regions. We aimed to summarize the role of these essential metals in TBI and its possible link with consciousness alterations. We found that TBI alters many neuronal molecular mechanisms involving essential metals, causing neurodegeneration, neural apoptosis, synaptic dysfunction, oxidative stress, and inflammation. This final pattern resembles that described for Alzheimer’s disease (AD) and other neurological and psychiatric diseases. Furthermore, we found that amantadine, zolpidem, and transcranial direct current stimulation (tDCS)—the most used treatments for DoC recovery—seem to have an effect on essential metals-related pathways and that Zn might be a promising new therapeutic approach. This review summarizes the neurophysiology of essential metals in the brain structures of consciousness and focuses on the mechanisms underlying their imbalance following TBI, suggesting their possible role in DoC. The scenario supports further studies aimed at getting a deeper insight into metals’ role in DoC, in order to evaluate metal-based drugs, such as metal complexes and metal chelating agents, as potential therapeutic options.

## 1. Introduction

DoC consist of an alteration in the mechanisms underlying wakefulness and/or awareness. Based on the degree of impairment of systems related to wakefulness and awareness, DoC can be classified into coma, unresponsive wakefulness syndrome (UWS), and minimally conscious state (MCS).

**Figure 1:**
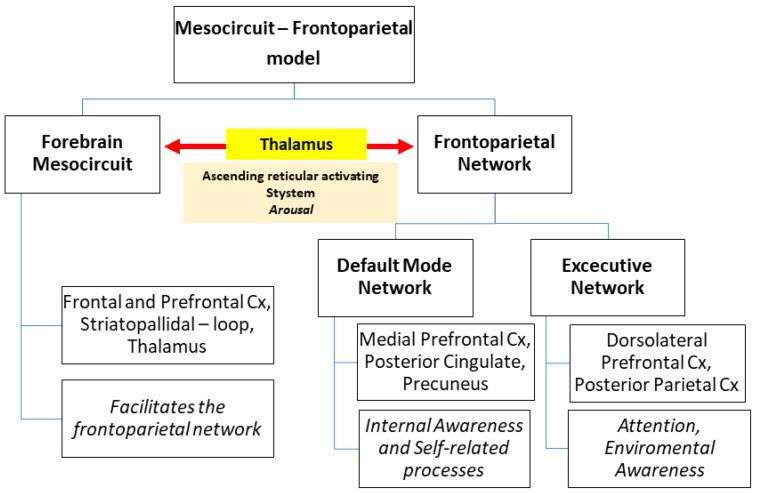
Mesocircuit—Frontoparietal Model. The diagram shows the interrelationship between the forebrain mesocircuit, the frontoparietal network, and the ascending reticular activating system. The function of each system is reported in italics. Cx: cortex.

Wakefulness depends on the proper functioning of an intricate system of serotonergic, dopaminergic, adrenergic, and cholinergic neurons located mainly in the brainstem: the ascending reticular activating system (ARAS) [1]. ARAS diffusely projects to the thalamus and cerebral cortex. It facilitates the proper and synchronous functioning of the anterior forebrain mesocircuit and the frontoparietal network, both of them essential for awareness [2] (Figure 1). In particular, the anterior forebrain mesocircuit includes the frontal and prefrontal cortex, striatopallidal—loop, and thalamus, and plays a key role in facilitating the frontoparietal network [3]. The frontoparietal network is divided into the default mode network and the executive network. The default mode network comprises regions from the medial prefrontal cortex, posterior cingulate cortex, and precuneus, and it is essential for internal awareness and self-related processes [4]. The executive network depends on regions from the dorsolateral prefrontal cortex and posterior parietal cortex, and it is central for attention and environmental awareness [5].

ARAS dysfunction can be caused by toxic or metabolic conditions, hemorrhage, and mechanical damage, alone or in combination, or by any condition that increases intracranial pressure that results in the decreasing of the oxygen supply. One of the most important causes of DoC is traumatic brain injury (TBI); other causes, such as ischemic stroke, intracranial hemorrhage, cardiac arrest, and others, are generally classified as non-traumatic brain injury (nTBI) [6]. In recent years, the important role of the essential metals Zinc (Zn), Iron (Fe), and Copper (Cu) in ARAS dysfunction has been emerging. This line of research is still in its infancy, but can take advantage of the knowledge gathered from other forms of brain damage or neurodegenerative disorders, as it is established that oxidative stress is strictly linked to essential metals imbalance and affects brain functionality. Zn, Fe, and Cu are either a trigger of oxidative stress, primarily in Fenton-type reactions, or co-factors and prosthetic groups of enzymes and proteins that regulate the brain function of the ARAS nuclei [1]. Herein, we will discuss Zn, Fe, and Cu involvement in ARAS and in DoC, with the aim to provide the reader with a concise description of the fundamental role played by these metals in TBI and their possible link with consciousness alterations.

## 2. Traumatic Brain Injury

### Traumatic Brain Injury: A Brief Description

TBI is a significant source of mortality and long-term disability worldwide, with an incidence of 69 million per year, and it is mostly due to falls and road accidents [7]. Following a head trauma, two kinds of brain injuries occur: primary and secondary [8].

It is not modifiable; the impact on the prognosis depends on the region affected and the strength and depth of force applied. Macroscopic lesions—such as focal cerebral contusions; intraparenchymal hematomas; and epidural, subdural, subarachnoid, and intraventricular hemorrhages—can occur as a result of direct damage to brain parenchyma or brain vessels. On the other hand, shearing forces depending on acceleration/deceleration mechanisms can cause microscopic axonal damage, with primary axotomy or progressive axonal dysfunction and delayed axonal degeneration, a condition defined as diffuse axonal injury (DAI) [9,10]. Secondary injuries develop immediately after the injury and continue over the following weeks. They consist of subtle, delayed, and cascading processes following primary trauma, such as excitotoxicity with neuronal membrane damage, synaptic dysfunction, neuronal swelling, mitochondrial impairment, inflammation, apoptosis, and ischemia, which can further exacerbate the primary injury [11,12]. A direct effect of TBI is the alteration in cerebral blood flow, with increased intracranial pressure and vasospasm that increase the risk of hypo-perfusion phenomena, which in turn can lead to cerebral ischemia and subsequent increased morbidity and mortality [9,10]. Many of the aforementioned features are similar to those observed in acute stroke [13,14] and in neurodegenerative disorders [15] where the imbalance of the essential metals Zn, Fe, and Cu plays an important role. Zn is primarily part of Zn finger proteins that are involved in DNA recognition [16], RNA packaging, transcriptional activation, regulation of apoptosis, protein folding and assembly, and lipid binding. Zn is also part of the Cu, Zn superoxide dismutase (SOD-1) that in mitochondria is vital to scavenging oxidative stress. The Fe bond to heme facilitates hemoglobin to carry oxygen in the blood; it is a component of cytochromes and of Fe–sulfur (FeS) proteins containing FeS cluster ligands [17], whereas the Cu of complex 4, cytochrome c oxidase [18], catalyzes the transfer of electrons in oxidative phosphorylation in mitochondria. All of them are required for energy production.

## 3. Essential Metals in Physiology

### 3.1. Zinc in Human Physiology at a Glance

As an essential trace element, Zn is required for the function of over 2000 metalloenzymes/proteins. It serves as a crucial component in the regulation of DNA and RNA biosynthesis, in hormone–receptor interactions, and in intracellular signaling, especially for neurotransmission, neurogenesis, or neuronal growth (reviewed in [19,20]). In 1963, the essentiality of Zn in humans was discovered [19], revealing that Zn deficiency is a worldwide concern related to malnutrition.

Zn content in the body (for a 70 kg adult male) is about 2.5 g; most of the body’s Zn is stored in skeletal muscle, bone, the liver, and the brain, while serum Zn accounts for less than 1% (10–15 µmol/L) [20]. 

Zn is absorbed in the small intestine by 2 families of transporters: Zrt/Irt-like proteins (ZIP), a family of 14 proteins, and ZnT, a family of 10 members [21,22,23]. ZnT and Zip transporters have opposite roles since Zip transporters increase cytoplasmic Zn concentrations, while ZnTs decrease them, e.g., ZnT1 is located in the small intestine and regulates Zn release from the enterocyte to the general circulation. 

Metallothioneins (MTs) are cysteine-rich intracellular proteins that bind metals. They are ubiquitous, and in the intestine’s mucosal cells, they can bind Zn and facilitate its excretion through its loss as cells slough off. In fact, Zn excretion occurs mainly through the intestine, in the pancreatic secretions, while it is lowered through urinary loss and the shedding of epithelial cells.

In serum, Zn^2+^ travels tightly bound to α2-macroglobulin (α2m) and loosely to albumin (Alb) and other proteins, peptides, and amino acids, which serve as a primary source of Zn accessible to all cells. Zn in serum is sharply reduced after severe trauma and inflammation, probably due to the simultaneous increase in the trans-capillary escape rate of α2m and Alb and the increased rate of their catabolism [24]. The decrease in circulating Zn levels during an acute phase can also be explained by the increased demand for the metal by the liver [25,26,27]. In the hepatocyte, Zn facilitates the biosynthesis of acute phase reactants and other essential processes, such as regulation of gluconeogenesis [28,29] in complex processes orchestrated by cytokines, critical modulators of inflammation [28,30,31]. 

Zn plays a central role in brain metabolism. It reaches the brain and crosses the brain capillary endothelial cells (BCECs) that form the Blood–Brain Barrier (BBB), reaching the neurons in the interstitial space. It enters the cerebrospinal fluid (CSF), a biological fluid secreted by the choroid plexus, and surrounds the brain, filling the brain ventricles. Zn is necessary for neurons’ growth and functioning. Transmembrane ZiP proteins facilitate Zn^2+^ ions’ transportation into neurons. Intracellular levels of Zn are regulated by ZnT family members that control Zn^2+^ trafficking and accumulation into vesicles. Zn is enriched into presynaptic vesicles by ZnT3, which loads Zn into glutamate synaptic vesicles and is released into the synaptic cleft together with glutamate. Among the most abundant Zn transporters, ZnT1, -3, and -6 regulate Zn brain levels. Its homeostasis is also controlled by the MT-3 protein that is exclusively expressed in the brain and plays a role in sequestering Zn in synaptic vesicles [32].

### 3.2. Iron in Human Physiology at a Glance

Fe is the most abundant trace element in our body, as we need it for oxygen supply and to convert blood sugar to energy. Furthermore, Fe helps the immune system, cognitive, and connective tissue functionality. We absorb only as much Fe as we need, on average 1–2 mg/day (reviewed in [33]). Fe absorption is, in fact, highly regulated, and the metal is not excreted, but recycled; our body loses some Fe only by indirect processes (e.g., sloughing of skin, minor hemorrhages, and menstruation in women), but in very little amounts (about 1–2 mg/day). The mainstream Fe enters as heme-Fe, and the remaining as inorganic, non-heme Fe. Our body contains almost 4–5 g of Fe that is distributed as follows: about 2.5 g in hemoglobin; 600 mg in reticuloendothelial macrophages; 300 mg in mitochondria proteins for cellular processes or energy production; and 3–4 mg in transferrin, traveling in the bloodstream—the residual is stored in ferritin [34].

Most all Fe is reduced to Fe^2+^ by reductases before entering the enterocyte. Divalent metal transporter (DMT1) facilitates Fe^2+^ transport within the cell. The Fe^2+^ can be absorbed as heme through the heme carrier protein 1 (HCP1) present on the apical surface of the enterocyte. A heme–oxygenase splits Fe^2+^ from heme and allows it to be stored in ferritin. Ferroportin located in the basolateral enterocyte surface transports Fe^2+^ to the portal plasma. Fe^2+^ is then oxidated to Fe^3+^ by the ferroxidase hephaestin (HP); Fe^3+^ binds to apo-transferrin, forming holo-transferrin (Tf), which transports Fe^3+^ in the blood. Fe levels in the blood are regulated by hepcidin, a hormone that is biosynthesized in the liver. Hepcidin regulates the degradation rate of ferroportin in the enterocyte membrane, and, as a result, the rate of Fe export from the enterocyte to the blood (reviewed in [33]).

In the liver, Fe is taken up by hepatocytes through endocytosis mediated by the transferrin receptor1 (TfR1). Fe reaches the mitochondria, where it is used for the biosynthesis of heme and Fe–sulfur clusters or for storage in ferritin, the main Fe reserve in the body. 

Fe^3+^ moves into the brain, transported by transferrin via the brain capillaries. By binding to the TfR1, it crosses the BCEC that forms the BBB, reaching the neurons in the interstitial space.

The TfR1 on the neuron surface mediates Fe endocytosis. Presynaptic vesicles bear ferroportin, suggesting that Fe^2+^ can travel via the synaptic vesicles to the synaptic cleft, where Fe^2+^ is released from the vesicles.

Glycosylphosphatidylinositol (GPI)-anchored ceruloplasmin (Cp-GPI) on the outside of the tip of astrocytes facilitates the oxidation of Fe^2+^ to Fe^3+^, allowing Fe uploading into apo-transferrin.

### 3.3. Copper in Human Physiology at a Glance

The role of Cu in human biology is essential: it serves as a protein cofactor in basic redox reactions in energy production involving cellular respiration, as well as free radical defense, collagen structure, neurotransmitter function, and Fe metabolism. Cu is absorbed in the small intestine into the enterocyte, and a pool of low-molecular-weight, soluble Cu^2+^ complexes is reduced by reductases to Cu^1+^ that is imported by CTR1. Within the enterocyte, the Cu-transporting P-type ATPase (ATPase7A) pumps Cu^2+^ out of the basolateral membrane via the vesicular compartment. Cu^2+^ then travels in serum to the liver through the portal vein, mostly bound to amino acids, peptides, micronutrients, and Alb as a pool of low molecular-weight Cu, known as non-ceruloplasmin (non-Cp) Cu.

It is then absorbed by the hepatocyte, through the CTR1. In the hepatocyte, ATPase7B, the homolog of enterocytes’ ATPase7A, incorporates Cu into ceruloplasmin that tightly binds 75–95% of Cu, while the residual 5–15% loosely binds to and is exchanged among Alb, α2m, amino acids, peptides, and several micronutrients (non-Cp Cu). Hepatocytes regulate non-Cp Cu levels in the blood to 0.008–1.6 µmol/L (after an overnight fast) [35]. Cu excess prompts ATPase7B to move from the trans-Golgi network to the canalicular membrane (via a vesicular compartment), where it mediates the release of the metal into bile.

Cu travels to the brain capillaries, mainly as non-Cp Cu, where it crosses the BBB [36], reaching CSF, where it has values in the range of 0.5–2.5 µmol/L. In the choroid plexus, Cu is taken up, mainly as non-Cp Cu from the blood, and is then released into the brain by processes mediated by CTR1, ATPase7A, and ATPase7AB [36].

## 4. Metal Involvement in the System of Serotonergic, Dopaminergic, Adrenergic, and Cholinergic Neurons Located in the Brainstem System Essential for Wakefulness: The Ascending Reticular Activating System

ARAS is an extensive network of more than 20 nuclei in each cerebral hemisphere and of interconnecting fibers, including the ‘diffuse modulatory systems’. They include noradrenergic neurons of the *locus coeruleus*, serotonergic neurons of the *raphe nuclei*, cholinergic neurons of the brainstem and basal forebrain, and the dopaminergic neurons of the *substantia nigra* and of the ventral tegmental area. The neurons of the diffuse modulatory systems have extensive and divergent axon projections, and a single neuron can make contact with 100,000 post-synaptic neurons, releasing neurotransmitters into the extracellular fluid that diffuses to numerous neurons. The diffuse modulatory systems of the ARAS control the rhythms of the thalamus.

Dopamine, noradrenalin (catecholamines), and serotonin play a central role in the cortical–subcortical circuitry of ARAS that takes part, alongside the role in consciousness, either in the regulation of mood, emotions, and sexuality or in cognitive function, primarily in executive functions, as well as in the regulation of sleep and appetite [37,38].

Cu (II) and serotonin have a potential risk of toxicity via oxidation of the serotonin and formation of compounds that are assumed to be unfavorable for neuronal survival [39]. 

In cells, including neurons, DihydrOxyPhenylAlanine (DOPA) is synthesized from tyrosine, and it is then used as a substrate by tyrosinase, a Cu-bearing enzyme, to catalyze the synthesis of melanin. The isomer L-DOPA is produced in neurons by tyrosine hydroxylase, a Fe-containing enzyme, and is converted to dopamine by the enzyme L-dopa-decarboxylase. The balance of the catecholamine is regulated by the enzyme beta-hydroxylase, which facilitates the synthesis of norepinephrine from dopamine [40]. Finally, monoamine oxidase (MAO) controls catecholamine hydrolysis.

Cu is a component of dopamine β-hydroxylase and MAO, and Fe of tyrosine hydroxylase, all involved in the catecholamine balance [40] that is altered in disorders of Cu metabolism, e.g., in Wilson disease, the paradigm of non-Cp Cu disbalance [35,41], and in a specific subtype of Alzheimer’s disease (AD), the main form of dementia in the elderly, namely, the ‘CuAD’ [42], typified by non-Cp Cu values higher than normal reference values (>1.6 µmol/L) [35,43].

## 5. Zinc, Iron, and Copper in Traumatic Brain Injury

### 5.1. Zinc Dynamics in Traumatic Brain Injury 

In the brain, Zn is mainly present in the hippocampus, neocortex, amygdala olfactory bulbs, and hypothalamus [44], in structural, mostly bound to proteins, and labile, 15–30% forms placed in the synaptic vesicles of glutamatergic glycine- and ϒ-aminobutyric acid-A (GABA)-ergic neurons [45,46,47,48,49].

**Figure 2:**
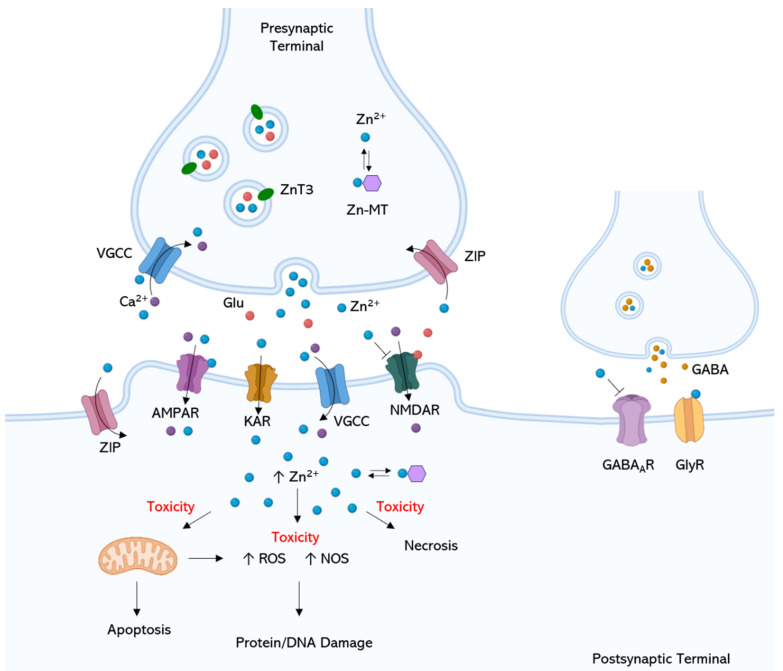
Physiological and pathological neuromodulation of Zn-containing pre- and postsynaptic neurons. Most vesicular Zn co-localizes with glutamate in subsets of glutamatergic zinc-enriched neurons, and it is also contained in the synaptic vesicles of subpopulations of glycinergic and GABAergic neurons. Zn^2+^-level regulation between cellular compartments, organelles, and extracellular space is ensured by ZIP and ZnT protein families, and by metallothioneins (MTs), which buffer cytoplasmic Zn^2+^, functioning as a temporary store for cellular Zn^2+^. In the presynaptic terminals, Zn^2+^ is transported into presynaptic vesicles by the Zn transporter ZnT3. During synaptic transmission, free Zn is released in the synaptic cleft, where it may be recycled back into the presynaptic boutons by ZIP/Zn^2+^ transporters or modulate excitatory (NMDA, AMPA) and inhibitory (GABA, glycine) amino acid receptors of the postsynaptic terminal; Zn can inhibit GABAAR and NMDAR, and potentiate/inhibit AMPAR and GlyR at low/high concentrations, respectively. Extracellular Zn can also alter the excitability of neurons through effects on voltage-gated ion channels (e.g., VGCC), affecting ions’ influx and neurotransmitter release. Ion channels and AMPAR/KAR allow synaptically released Zn to enter presynaptic and postsynaptic neurons to modulate intracellular Zn signaling functions. Excessive Zn^2+^ accumulation inside postsynaptic cells, as per excitotoxic stimulation, can lead to a series of toxic effects involving mitochondrial dysfunction and ROS/NOS production, eventually leading to oxidative damage to proteins and DNA, neuronal apoptosis, and/or necrosis. Glu, glutamate; GABA_A_R, GABA A receptor; NMDAR, NMDA receptor; AMPAR, AMPA receptor; GlyR, glycine receptor; VGCC, voltage-gated calcium channel; KAR, kainate receptor.

Zn ions are enriched into presynaptic vesicles by the ZnT3 transporter and released into the synaptic cleft upon neuronal activity [50]. Upon release, free Zn in the synaptic cleft can bind and modulate pre- and postsynaptic receptors and channels, including the glutamate receptors N-methyl-d-aspartate (NMDAR), α-amino-3-hydroxy-5-methyl-4-isoxazolepropionic acid (AMPA), and kainate receptors, glycine, GABAA receptors, and voltage-gated calcium (Ca) channels [51,52,53]. In turn, Zn bound to proteins mainly acts as a reservoir of Zn in the synapse; proteins, such as metallothioneins (e.g., brain MT-3) and Zn-finger proteins, can bind to Zn and regulate its availability in the synapse and in different regions of the brain. Zinc bound to proteins also regulates the activity of enzymes, transcription factors, and signaling molecules, affecting synaptic transmission and plasticity [32,44,54] (Figure 2).

Early work revealed toxic effects of Zn in TBI, mainly related to oxidative stress burst, excitotoxicity, and mitochondrial dysfunction, eventually leading to neuronal apoptosis and/or necrosis (reviewed in [55]). As Ca, Zn can partake in NMDAR-mediated excitotoxicity, eventually leading to cell death [49]. Intraneuronal release of Zn^2+^ may also impair mitochondrial functioning [56]: the metal can prompt the permeabilization of the mitochondrial membrane through the activation of the mitochondrial permeability transition pore that facilitates the release/production of proapoptotic factors, such as cytochrome c, and the apoptosis-inducing factor [57] (Figure 2). 

Seminal studies revealed [51,52,53] that within the first 6 h after injury, Zn^2+^ is released from presynaptic buttons and is associated with glutamate excitotoxicity processes that lead to postsynaptic neuronal death [51,58]. Zn displacement in these first few hours after injury is the result of rapid transfers from synaptic vesicles, from binding metallothioneins MT-3 in the brain, and from mitochondrial Zn stores, leading to increased cytoplasmic Zn^2+^, which is toxic [51,52,53,55,58]. Upon presynaptic buttons release, Zn appears in the cell bodies of injured postsynaptic neurons; free Zn appears in damaged somata neurons and penetrates the somata through both voltage and ligand-gated ionic channels [51]. A subsequent study revealed the appearance of Zn^2+^ in injured postsynaptic neurons also at 24 h, then again at 7 days [58]. Chelating agents, such as CaEDTA, reduced the number of damaged neurons after TBI in the CA3 region of the hippocampus, but only if the treatment occurred within the first 6 h after injury [59,60]. Chelating treatments at later times had deleterious effects; chemical blocking of vesicular Zn ions worsened the effects on the aftermath of TBI by increasing the number of necrotic and apoptotic cells within the first 24 h after TBI [61], suggesting the beneficial effect of Zn in the reactive processes that follow the acute phase, as will be discussed later.

### 5.2. Iron Dynamics in Traumatic Brain Injury

Compared with Zn, the number of studies on the involvement of Fe and Cu is small, although the clinical significance of these two transition metals in both primary and secondary TBI reactive processes is unquestionable, as demonstrated primarily in studies targeting neurodegeneration. Experimental models of TBI reveal that Fe increases in the acute phase (6 h after injury) and has a major increase in the region closest to the lesion [58].

Subsequent increases have been revealed at 72 h, then at 7 and 14 days, as well as a maximum increase at 28 days with a concomitant increase in ferritin [58].

The amount of red blood cell residual from a cerebral hemorrhage in TBI is the greatest source of Fe deposition in the tissue. It causes brain injury: heme oxygenase 1 (HO-1) catalyzes heme oxidation and Fe release from red blood cells, then free Fe^2+^ can trigger oxidative stress [58,62,63] via the Fenton reaction. Free Fe^2+^, heme, hemoglobulin, and other blood-derived products are potent cytotoxic agents that can boost oxidative stress, inflammation, and cell signaling disruption, eventually leading to cell death (reviewed in [64]). Further free Fe^2+^ is released by microglia that engulf red blood cells and release free Fe^2+^ into the interstitial space of the brain [65]. Dysfunction in mitochondria and lysosomes occurs in TBI and can account for severe oxidative injury within the cell. A large amount of Fe and Ca ions move in, disrupting the normal function of the mitochondria, primarily via ROS generated by Fe through the Fenton reaction [66]. An important contribution to TBI damage is also provided by the release of Fe^2+^ contained in lysosomes: free Fe^2+^ pooling in the cytoplasm is the most powerful producer of reactive oxygen species (ROS) in cells [67]. Fe accumulation and overload in the site of injury can also change the size and number of lysosomes, affect autophagic flux, and cause autophagic cell death [68]. Increased free Fe^2+^ and glutamate overload as a result of excitotoxicity phenomena activation in the synaptic cleft may inhibit glutathione (GSH) synthesis and then facilitate ferroptosis, a Fe-dependent cell death characterized by GSH depletion and a build-up of lipid peroxides that results in cell death (reviewed in [13]). The increase in Fe at 7, 14, and 28 days, as has been revealed in experimental animal models [58], is associated with secondary injury processes related to head trauma that include the release of blood metabolites, microglial activation, thrombin activity, and proinflammatory factors, contributing to the final severity and recovery of nerve injury after TBI [69]. In fact, the accumulation of Fe and ferritin, the Fe handling protein, has been observed greatly distal to the cortical injury site in the brain after injury [58,62]. In humans, once the acute phase is over, the series of secondary injury cascades of TBI can still lead to a poor prognosis. Metal-altered metabolism, and in particular Fe accumulation in tissues, is also part of mild traumatic brain injury (mTBI) that is often ignored because its initial symptoms do not seem serious. By using magnetic field correlation MR imaging in humans that is sensitive to the presence of non-heme Fe, Fe accumulation has been demonstrated at sub-thalamic regions greatly distal to the cortical site of injury [63].

Studies in humans revealed that decreased serum transferrin and Fe and increased ferritin were associated with severe cerebral edema volume and with a poor prognosis [70].

### 5.3. Copper Dynamics in TBI

Experimental models of TBI [58] show that Cu concentrations increased in the ipsilateral cortex adjacent, but not closest, to the impact zone only 28 days after the injury. If, on the one hand, this elevation might be related to Cu-dependent processes of demyelination or remyelination, on the other hand, it may be cause for concern in relation to the potential chronic oxidative stress toxicity based on Cu abnormal metabolism, as it has been observed in neurodegenerative disorders and more specifically for AD.

Elevated intracranial pressure (ICP) is a major secondary pathology after TBI and a major contributor to morbidity and mortality. Elevated ICP is defined as a measurement of 25 mmHg for at least 5 min that is verified twice in a 24 h period or on 2 consecutive days. Lower serum ceruloplasmin levels within the first 24 h after trauma are prognostic of elevated ICP in patients with TBI. A cut-off of 14 mg/dL had a sensitivity of 87% and specificity of 73% for identifying patients who developed ICP.

Similarly, a low serum total Cu level (less than 20.76 µmol/L) was also predictive of high ICP (sensitivity 86%, specificity 73%). Three days after the injury, ceruloplasmin levels increased.

This dynamic resembles the Cu dynamics after Myocardial Infarction (MI) [71,72], demonstrating an 80% release of Cu from the damaged myocardial tissue caused by myocardial infarction and a subsequent uptake of Cu by the liver; the increase in serum ceruloplasmin at 3 days in response to myocardial infarction was the result of the increased Cu uptake of serum non-Cp Cu released from the damaged myocardium, rather than resulting from an inflammatory response.

Cu is a particularly potent pro-oxidant and can form hydrogen peroxide and subsequent hydroxyl radicals at a high rate, significantly higher than that of Fe [58].

## 6. TBI and AD Share Neuropathological Processes Involving Metals Imbalance

### 6.1. TBI and AD Share Neuropathological Processes

Beta-amyloid (Aβ), alpha synuclein (α-syn), and hyper-phosphorylated tau are some of the most frequently reported molecules upregulated in TBI and are also closely associated to AD [73].

Aβ overproduction and accumulation in brain tissues are known to induce synaptic alterations and neurodegeneration [74,75]. Aβ peptides are generated via the trans-membrane cleavage of amyloid precursor protein (APP) by β- and γ-secretases, specifically by beta-site APP cleaving enzyme (BACE) and the catalytic component of β-secretase, presenilin-1 (PS1), within the axonal membrane compartment [76,77,78,79,80]. The disruption of axonal transport after TBI creates an environment characterized by extensive co-accumulation of APP with its cleavage enzymes BACE and PS1, leading to Aβ peptide production [81,82]. Recently, a shift from a “neuronal” to a “neurovascular” point of view has been proposed to explain Aβ accumulation in TBI, since the vascular shear stress characterizing TBI can induce acute BBB disruption, contributing to ischemic damage, hypoperfusion, vascular dysfunction, and consequent Aβ deposition [83,84,85]. Oxidative stress and neuroinflammation can lead to overproduction of Aβ, and Aβ in turn can induce oxidative stress and damage [74], creating a vicious circle. Different mechanisms by which Aβ mediates oxidative damage are described, including interference with the normal mitochondrial function and ROS production and removal [86,87]; dysregulation of metals homeostasis with increased levels of Fe, Cu, and Zn in AD patients [88,89,90]; and stimulation of neuroinflammation through the production of inflammatory cytokines and chemokines [91,92], oxygen free radicals [93], nitric oxide [94], and tumor necrosis factor α [95], further promoting Aβ accumulation and neurodegeneration.

The other classic pathological findings in AD are neurofibrillary tangles (NFTs) containing abnormal forms of the microtubule-associated protein tau. Following TBI, hyperphosphorylated tau has been found in brain tissue, as well as CSF [96,97]. Recently, it has been reported that repetitive axonal injury in TBI initiates a series of metabolic, ionic, and cytoskeletal disturbances that trigger a pathological cascade, leading to chronic traumatic encephalopathy (CTE) characterized by the accumulation of P-Tau in neurons and astrocytes in a pattern that is distinct from other tauopathies, including AD [98]. The whole process leading to tau accumulation after TBI is still unclear; however, it has been proposed that mechanical stress can disrupt microtubule networks within axons, leading to diffuse axonal injury, tau release, hyper-phosphorylation, and extracellular accumulation [99,100]. Oxidative stress is also well recognized in tauopathies where abnormal forms of tau protein accumulated [101]. Mitochondrial dysfunction leading to excessive ROS production, impaired bioenergetics, and transport along the neuronal axon causes tau hyperphosphorylation and neurofibrillary tangles accumulation by regulating the activity of protein kinases and phosphatases [102]. Furthermore, aberrant tau may induce ROS production and oxidative stress by promoting more mitochondrial impairment [103]. Trauma-induced cerebrovascular dysfunction can also contribute to tau release, hyperphosphorylation, and accumulation following TBI [104]. Thus, cerebrovascular pathology appears a mechanistic link between TBI and AD since neurovascular injuries can accelerate Aβ synthesis and accumulation on the artery wall, while arterial stiffness, α-syn protein misfolding, persistent inflammation, tau hyperphosphorylation, and tau/Aβ-induced BBB damage concur in post-concussive syndrome and dementia (recently reviewed in [15,105]).

Prion protein (PrP) is another molecule proposed to have a link between TBI and AD. PrP is a cell surface glycoprotein highly expressed in the central nervous system, especially in neurons [106]. Neuronal functions of PrP include metal binding activity as Cu buffer, pro- and anti-apoptotic roles, cell signaling, neuronal morphology, cell adhesion, and maintenance of oxidative stress homeostasis [107] and long-term memory [108]. In brain injury, PrP has been connected with TBI-associated memory impairment [109], together with enhanced deposition in brain tissue and memory loss [110]. Plasma PrP levels have also been proposed as a potential biomarker for TBI [111].

PrP has recently been linked to AD neuropathology [112]: it has been shown to co-localize with Aβ in plaques [113] and to function as a high-affinity receptor for Aβ oligomers triggering the Fyn-mediated intracellular signaling cascade [114,115]. This mechanism also contributes to the hyperphosphorylation and mislocalization of Tau protein [116], producing NFTs and the cognitive impairment of AD.

### 6.2. Iron and Copper Involvement in AD

Studies around 50–60 s [117,118] have firstly shown Fe involvement in AD, mainly associated with plaques, tangles, and microglia. A pivotal phase II clinical trial with the Fe chelator deferoxamine in the 1990s demonstrated a slowing of the progression of AD [119]. However, this phase II study had some weaknesses, which included that it was not double-blind and that it was designed (proof of concept) to reduce aluminum toxicity, while Fe was administered during treatment. Furthermore, it is known that although DFO has great potential to mitigate Fe accumulation in clinical settings, its widespread use is limited by adverse side effects. Treatment with deferoxamine is in fact associated with numerous systemic toxicities, which include impairment of renal and hepatic function, ototoxicity, and ocular and nervous system toxicity, as well as toxicity to the respiratory, cutaneous, cardiovascular, and gastrointestinal systems (review in [120]).

Then levels of Fe, transferrin, and ferritin were shown to be abnormal in the AD brain [121,122], and Fe was seen to be enriched in neurofibrillary tangles and Aβ plaques made of tau proteins and Aβ peptides, respectively. Seminal studies revealed that Fe was the source of oxidative stress of these pathognomonic findings of AD [123,124,125,126], as also confirmed by more recent studies showing the Fe distribution in the AD brain areas most damaged by the disease [127,128].

A direct link of Fe in AD pathology has been revealed by studies investigating changes of Fe status and Aβ plaques formation [129,130,131]. Fe regulates the APP cleavage in which APP is first cut by β-secretase at a site placed outside the cell surface and then by γ-secretase in the transmembrane domain, producing the Aβ peptide (reviewed in [132,133]). Thus, Fe enables Aβ peptides synthesis, facilitating its aggregation and plaques formation [129,130,131]. Furthermore, Fe exposure was shown to induce the expression of APP [134], while APP has been reported to increase the Fe export protein ferroportin and facilitate the release of Fe from neurons [135]. Tau protein facilitates APP coupling with ferroportin on the cell surface to export Fe [136].

Previous genetic association studies revealed that the hemochromatosis risk allele H63D was associated with an increased risk of AD [137,138]. The H63D gene variant has been associated with clinical features of AD, e.g., the disease onset [139,140], cognitive symptoms [141], severity of clinical deficits [142], AD markers in the CSF [143], and conversion from mild cognitive impairment (MCI) to AD [141]. However, a more recent study did not confirm such an association and suggested that previously reported associations were the result of more complex interactions or were limited by the small size of the sample [144].

Increased CSF ferritin and ceruloplasmin levels [145,146,147,148,149], and Fe accumulation burden as revealed by neuroimaging [150,151] or directly measured in post-mortem brain specimens [152], appeared to predict longitudinal cognitive impairment and brain atrophy in individuals with underlying AD pathology, thus demonstrating the Fe burden association with AD progression, even though Fe serum markers fall in the normal reference range (reviewed in [152]).

The underlying pathological mechanisms linking altered Fe metabolism to neurodegeneration include APP cleavage (reviewed in [132,133]) and Aβ peptide production (reviewed in [41,42]), along with a critical role of Fe in accelerating the aggregation of tau proteins in neurofibrillary tangles [124,126].

Fe, in the Fe^2+^ form, catalyzes the Fenton reaction: it mediates the production of one of the most reactive ROS species, that is, the hydroxyl radical •OH, strongly increasing oxidative stress and inflammatory processes by microglia (reviewed in [33]).

Furthermore, Fe in the brain tissue might be harmful through ferroptosis (reviewed in [13,152]), a Fe-dependent cell death [153], typified by glutathione (GSH) depletion and a rise in lipid peroxidation, which induce toxicity, eventually leading to cell death [153].

Experimental models of TBI [58] show that Cu concentrations were increased in the ipsilateral cortex adjacent, but not closest, to the impact zone only at 28 days after the injury, and it may be cause for concern in relation to the potential chronic oxidative stress toxicity based on Cu abnormal metabolism, as has been observed in AD.

**Figure 3:**
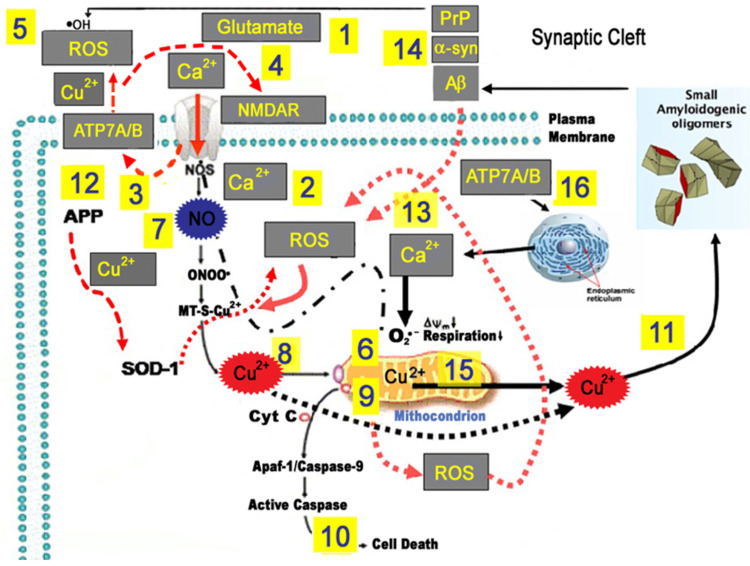
Model of Beta amyloid (Aβ), glutamate, oxidative stress, and ionic dyshomeostasis in neurodegenerative processes associated with Traumatic Brian Injury (TBI) and Alzheimer’s disease (AD). Aβ, alpha synuclein (α-syn), and hyper-phosphorylated Tau are among the most frequently reported molecules upregulated in TBI and are also closely related to AD. Experimental models of TBI [58] show that Cu concentrations were increased in the ipsilateral cortex adjacent, but not closest, to the impact zone only 28 days after the injury, and it may be cause for concern in relation to the potential chronic oxidative stress toxicity based on Cu abnormal metabolism, as has been observed in neurodegenerative disorders and more specifically for AD. The model proposed to highlight the main Cu toxic mechanisms that can be triggered by TBI in the long term and in AD. In a complex scenario, Aβ, oxidative stress, excitotoxicity, and Cu^2+^ dyshomeostasis act in concert to promote synaptic dysfunction and neuronal loss. Upon the production of excessive glutamate levels (1), Ca^2+^ ions enter the cell through the NMDA receptor (2) and (3) induce Cu-ATPase7A/B (ATP7A/B) translocation at synapses where vesicular Cu is released in the synaptic cleft. The released Cu^2+^ (in concentrations up to 100 µmol/L) may inhibit the NMDA receptor, thereby protecting neurons from glutamatergic excitotoxicity (4), or catalyze Fenton-type and Haber–Weiss reactions, thereby generating reactive oxygen species (ROS) (5). Enhanced ROS generation can damage proteins, lipids, and nucleic acids, eventually leading to cell death (5). Ca^2+^ overload can increase superoxide anion (O2•−) production from mitochondria (6), and nitric oxide (NO) generation via Ca^2+^-dependent activation of NO synthase (NOS) (7). Reactive oxygen and nitrosative (RNS) species mobilize Cu^2+^ from metallothionein 3 (MT-3) (8), leading to increased intracellular toxic Cu^2+^ concentrations (9) and promoting mitochondrial dysfunction, as well as release of pro-apoptotic factors (10). ROS-driven Cu^2+^ mobilization can further aggravate oxidative stress and initiate Aβ oligomerization (11). Altered trafficking of APP and/or elevated Aβ oligomer secretion can generate an intracellular Cu^2+^ deficiency, thereby causing oxidative stress by the loss of SOD-1 function (12). Aβ, α-synuclein, and PrP increased after TBI can modulate neurotransmission as [Cu^2+^] buffers within the synaptic cleft or amplify the vicious cycle by increasing oxidative stress (13). Furthermore, glutamate-driven mitochondrial Ca^2+^ overload can mobilize Cu^2+^ from these organelles (14). Excess Non-Cp Cu in the bloodstream is a source for the buildup of labile Cu^2+^ into the intermembrane space of mitochondria (15), promoting the ATPase7A/B translocation of Cu^2+^ into vesicles of the trans-Golgi network and endoplasmic reticulum (ER) (16). These processes, working intracellularly at the level of synaptic spines, in the synaptic cleft, and in the neurovascular unit (ref. [8]), can facilitate synaptic dysfunction, neuronal deafferentation, and ultimately brain cell death.

A sizable number of studies sustain a major role of Cu in AD, as summarized in the “Theory of metal imbalance in AD” [42,90]. This theoretical construct claims the existence of a disease-associated metabolic sub-pathway in AD, typified by a gradual shift of Cu from bound to proteins to pools of loosely bound metal ions, engaged in oxidative stress [42]. This theory is built on the evidence that a subpopulation of AD patients shows abnormal values of non-Cp Cu [90], which represents the main species of Cu loosely bound and exchanged among albumin, α1-macroglobulin, peptides, and amino acid in serum and is potentially toxic. The theory is based on massive evidence demonstrating that Cu has a direct role in Aβ aggregation and can cause neurotoxic effects by promoting deficits of intracellular Cu-bound proteins [154,155], since Cu bound to Aβ undergoes redox cycling reactions, with O2 prompting Fenton-type reactions [156]. Furthermore, mutations in genes of the Aβ pathway (including AβPP and PSEN1/PSEN2) affect the Cu-buffering AβPP/Aβ capacity [157,158]. The accumulated body of evidence (reviewed in [42]) partially supports the idea that metal dysregulation might be a crucial player also in the neurodegeneration associated with TBI (Figure 3). Metal ion imbalances, energy depletion of high-energy-demand neurons, oxidative stress, and protein misfolding associated with Cu imbalance might be dysregulated also in TBI and result in neuron death (Figure 3).

### 6.3. TBI and Psychiatric Disorders Share Neuropathological Processes

Psychiatric disorders occur commonly after TBI [159]. The regions more vulnerable to neurotrauma include different frontal–subcortical circuits with significant roles in cognition, executive function, and social behavior [160]. Thus, brain injury involving frontotemporal regions can worsen pre-existing mental illness or cause new psychiatric symptoms, including anxiety, depression, mood swings, anger, acute stress, obsessive–compulsive and psychotic disorders, and post-traumatic stress disorder (PTSD) [161].

Depressive and anxiety disorders, especially major depressive disorder and PTSD, showed the highest rates in individuals with TBI, usually emerging in the first year postinjury and with depression more persistent postinjury [161,162,163,164]. Damage to neuronal circuits in the prefrontal cortex, amygdala, hippocampus, basal ganglia, and thalamus was linked to post-TBI depression [159,165], along with alterations in the serotonin, norepinephrine, glutamate, acetylcholine, and dopamine neurotransmission systems, observed in animal models and TBI patients [165,166]. Hypoxia could also lead to the release of free radicals and excitotoxic neurotransmitters to cause further neuronal damage to these systems [167].

Alterations in the homeostasis of Cu, Zn, and Fe metals have been recognized as important in the development of neuropsychiatric disorders. Patients with depressive disorders showed a significantly elevated concentration of ceruloplasmin and Cu in their blood [168]. Cu acts as a cofactor of enzymes involved in the turnover of catecholamines and in the catalytic activity of some antioxidant enzymes, and it participates in oxidative and nitrosative stress processes [169,170,171,172]. Moreover, elevated levels of Cu inhibit the function of NMDA and AMPA receptors, thus disturbing glutamatergic transmission [173,174]. On the contrary, Zn deficiency had a strong link with depression and anxiety in patients, as well as in rodent models [175,176]. Zn has been found to regulate GABA and glutamate transmission and modulate GABAergic inhibition and impairment [177,178]. Zn deficiency was shown to enhance the expression of NMDA receptor subunits in the hippocampus and prefrontal cortex and to decrease brain-derived neurotrophic factor (BDNF) levels essential for normal neurotransmission and neuron survival [179,180]. Moreover, low intracellular Zn has been associated with DNA damage, oxidative stress, antioxidant defense, and DNA repair [181,182,183]. In addition, brain Fe deficiency was demonstrated to cause poor myelination and impairment of monoaminergic, glutamate, and GABA homeostasis, promoting deficits in memory and behavior, as well as anxiety, depression, and bipolar disorders [184,185,186,187].

## 7. Therapeutic Intervention for Recovery from Traumatic Brain Injury Disorders of Consciousness

### 7.1. Stimulating Consciousness Recovery: Amantadine, Zolpidem and Transcranial Direct Current Stimulation

At the moment, few evidence-based treatments for recovery from DoC are available. Among pharmacological treatments, amantadine is effective in enhancing consciousness recovery among TBI DoC patients [188]. Amantadine is an antiparkinsonian agent, and its mechanism is not fully understood. Amantadine appears to act as a NMDA and indirect dopamine agonist [189]. A recent study found that amantadine has some effects on Ca^2+^ fluxes in brain cells, facilitated by transient receptor potential cation channel subfamily V member 4 (TRPV4) and transient receptor potential cation channel subfamily M member 2 (TRPM2) [190]. TRPV4 encodes for a Ca^2+^-permeable, nonselective cation channel involved in the regulation of systemic osmotic pressure by the brain [190,191,192]. TRPM2 is a non-selective calcium-permeable cation channel of the Transient Receptor Potential ion channel superfamily thought to play a role in bipolar affective disorder [191] and in neuroblastoma [192]. It has been demonstrated that amantadine can reduce hypoxia-mediated mitochondria ROS, apoptosis, and TRPM2/TRPV4-mediated overload Ca^2+^ influx [190]. It has been reported to be neuroprotective in neurodegenerative and cerebrovascular diseases associated with the upregulation of mitochondrial ROS, Ca^2+^, and Zn^2+^ concentration [190]. Zolpidem is a hypnotic drug targeting a specific site of the GABA-A receptor, and it is considered a therapeutic choice in disorders of consciousness [193]. The role of zolpidem in essential metals-mediated ARAS pathways is unclear, although it seems to modulate post-hypoxia Ca-dependent pathways [194].

Regarding non-invasive brain stimulation treatments, transcranial Direct Current Stimulation (tDCS) is effective in enhancing consciousness recovery [195]. It is known from animal studies that tDCS can stimulate neural plasticity processes, causing NDMR activation and BDNF release [196]. Recent studies have pointed out that tDCS can modulate brain plasticity facilitating Fe-, Zn-, and Cu-mediated pathways in the nucleus accumbens, hypothalamus, and ventral tegmental area, with a consequent dopaminergic and serotonergic regulation [197].

### 7.2. Potential of Zinc in TBI: New Perspective on the Mechanisms Underlying Mortality, Medical Complications, and Consciousness

TBI activates Zn release from synapses in the early stages (6 h) [58], which may contribute to excitotoxicity, but Zn in later stages (1 month) may support neuronal regeneration (reviewed in [55]). Studies in animal models of TBI have shown that Zn treatment can improve learning and memory and can prevent mood disorders associated with TBI [198]. Zn treatment (intraperitoneal injection plus Zn supplementation) reduced depression-like behaviors in preclinical models [198,199] and other TBI-associated sequelae, suggesting that Zn before the injury may provide protection [198]. Furthermore, findings suggested a role of Zn in hippocampal neurogenesis: rats fed a Zn-supplemented diet for 4 weeks, followed by TBI, showed increases in the total density of newly born cells [198,199].

In humans, it has been seen that TBI increased urinary Zn excretion over 14-fold above normal, coupled with significantly decreased serum Zn levels [200], suggesting an increased bodily demand for the metal.

An early randomized, prospective, double-blinded, controlled trial of standard Zn therapy [201] enrolling 68 TBI patients for one month showed a decrease in the mortality rate and improvements in the Glasgow Coma Scale scores [201]. A more recent study (randomized placebo-controlled phase II study of 50 Zn patients taking 120 mg/day vs. 50 placebos) dated 2018 showed that the Organ Failure Assessment, Glasgow outcome score, inflammation factors, and length of stay were significantly improved in Zn patients with severe head injury with respect to placebo [202].

As a whole, Zn supplementation in TBI has been associated with an improved neurological recovery rate, a shorter period of hospitalization, and a borderline decrease in the mortality rate on day 28 ([201,202], reviewed in [55]), suggesting potential beneficial effects to curb the disease.

## 8. Conclusions

After TBI, Fe, Cu, and Zn are involved in a pathological cascade, causing neurodegeneration, neural apoptosis, synaptic dysfunction, oxidative stress, and inflammation. This trend resembles what is described for other neurodegenerative diseases, particularly AD. Compared to the other metals examined, Zn seems to have a possible neuro-regenerative role. Some therapeutic treatments with beneficial effects for DoC, including amantadine, zolpidem, and transcranial direct current stimulation, exert some of their actions in pathways related to essential metals. Furthermore, Zn therapy has apparently beneficial effects after TBI, both on the overall prognosis and consciousness, likely based on the neurogenesis properties of the metal. Further studies on the role of essential metals in DoC are needed in order to stimulate new research on potential innovative therapies.

## Data Availability

Not applicable.
